# Open-Set Recognition of Wood Species Based on Deep Learning Feature Extraction Using Leaves

**DOI:** 10.3390/jimaging9080154

**Published:** 2023-07-30

**Authors:** Tianyu Fang, Zhenyu Li, Jialin Zhang, Dawei Qi, Lei Zhang

**Affiliations:** 1 College of Mechanical and Electrical Engineering, Northeast Forestry University, Harbin 150040, China; 2 Dean’s Office, The Open University of Harbin, Harbin 150001, China; 3 School of Computer and Information Engineering, Harbin University of Commerce, Harbin 150028, China; 4 Department of Diagnostic Radiology and Nuclear Medicine, University of Maryland School of Medicine, Baltimore, MD 21201, USA; cszhanglei@gmail.com

**Keywords:** leaf open-set recognition, deep learning feature extraction, weighted SVDD

## Abstract

An open-set recognition scheme for tree leaves based on deep learning feature extraction is presented in this study. Deep learning algorithms are used to extract leaf features for different wood species, and the leaf set of a wood species is divided into two datasets: the leaf set of a known wood species and the leaf set of an unknown species. The deep learning network (CNN) is trained on the leaves of selected known wood species, and the features of the remaining known wood species and all unknown wood species are extracted using the trained CNN. Then, the single-class classification is performed using the weighted SVDD algorithm to recognize the leaves of known and unknown wood species. The features of leaves recognized as known wood species are fed back to the trained CNN to recognize the leaves of known wood species. The recognition results of a single-class classifier for known and unknown wood species are combined with the recognition results of a multi-class CNN to finally complete the open recognition of wood species. We tested the proposed method on the publicly available Swedish Leaf Dataset, which includes 15 wood species (5 species used as known and 10 species used as unknown). The test results showed that, with F1 scores of 0.7797 and 0.8644, mixed recognition rates of 95.15% and 93.14%, and Kappa coefficients of 0.7674 and 0.8644 under two different data distributions, the proposed method outperformed the state-of-the-art open-set recognition algorithms in all three aspects. And, the more wood species that are known, the better the recognition. This approach can extract effective features from tree leaf images for open-set recognition and achieve wood species recognition without compromising tree material.

## 1. Introduction

At present, spectroscopy, stoichiometry [1], image-processing methods based on traditional machine learning, and image-processing methods based on deep learning are the most widely used to recognize known wood species in the field of wood species recognition. In spectroscopy, Raman spectroscopy [2], fluorescence spectroscopy [3], and infrared spectroscopy [4] are mainly used to recognize wood species. In traditional machine learning-based image processing, gray-level co-occurrence matrix [5], basic gray-level aura matrix and statistical properties of pores distribution [6], fuzzy logic [7], kernel genetic [8], macroscopic image analysis and SVM [9], multidimensional texture analysis and SVM [10], improved basic gray-level aura matrix [11], and fuzzy logic and SVM [12] are used to recognize wood species. For deep learning-based image processing, convolutional neural networks are the best performing algorithms, and SqueezeNet [13], ResNet [14], and Kayu30Net [15] are used to recognize wood species. These methods are closed-set recognition algorithms, while the open-set recognition of wood species has been less studied. However, the ideal case of closed-set recognition rarely exists in reality. In open-set recognition [16], the training samples are all known classes (in distribution, ID), while the test samples are mostly unknown classes (out-of-distribution, OOD). In this situation, the task is to accurately recognize the species of known species, and to detect the species of unknown species. The use of tree leaves to recognize wood species can effectively reduce damage to the trees themselves, thus enabling true non-destructive testing. The abovementioned methods are closed-set recognition methods, and all require cutting wood material.

Currently, the algorithms and theoretical research frameworks that can complete the open-set recognition of wood species mainly include machine learning algorithms for anomaly detection/novelty detection (OC-SVM [17], SVDD/weighted SVDD [18], and Bayesian SVDD [19,20]). In recent years, some researchers have started to use advanced machine learning algorithms [21,22] and deep learning algorithms [23,24,25] for open-set recognition. Open-set recognition algorithms aim to identify unknown categories. There are two ways to construct the training set for current open-set recognition algorithms [26]. The first is to include only known categories, and the second is to include a small number of unknown categories. We used the former to construct the training set.

Currently, most of the leaf data for wood species are image data, and deep learning algorithms can efficiently extract the deep features of images, which is a method that has been well applied in various image recognition fields. In the field of wood, a deep learning algorithm [15] is used to recognize RGB images of the cross-sections of wood blocks, which is a closed-set recognition in these applications. The support vector description algorithm [18] is an excellent anomaly detection/novelty detection algorithm that can efficiently recognize unknown samples. However, the input dimension of the support vector description algorithm is one-dimensional data, while the image data are two-dimensional data, and corresponding rules and dimension reduction algorithms are needed.

In this study, we propose a novel method using a deep learning-based algorithm to extract features from images and using a traditional machine learning algorithm as the backend for single-class classification. After recognizing the known and unknown classes, the trained deep learning algorithm was used to complete the open-set recognition scheme for the leaf recognition of wood species with known classes. Leaf image data were recognized for 15 wood species. In our experiments, the recognition was performed under the condition that the two datasets were split. The first partition consisted of 5 known and 10 unknown wood species, and the second partition consisted of 10 known and 5 unknown wood species, with the main purpose being to verify the robustness of the experiment under different data partitions.

The contributions of the proposed scheme are as follows:

1. This scheme achieved the open-set identification of wood species and leaves. Currently, wood species recognition is mostly performed in closed sets. It is exciting to be able to achieve an efficient open-set identification without compromising wood species.

2. In this scheme, an open-set recognition framework for wood species based on deep learning feature extraction is proposed. In this framework, a trained CNN is used to extract the deep features of the image, and the weighted SVDD algorithm is used for single-class recognition and the known classes are recognized by the trained CNN. The results of single-class recognition and multi-class recognition are combined to realize open-set recognition.

In this paper, we combine the single-class classification algorithm and the multi-class classification algorithm to form an open-set recognition scheme. Experimental results showed that only the proposed method could perform the open-set recognition efficiently, while the state-of-the-art wood species classifiers failed. 

## 2. Materials and Methods

### 2.1. Leaf Dataset of Wood Species and Experimental Environment

**Figure 1:**
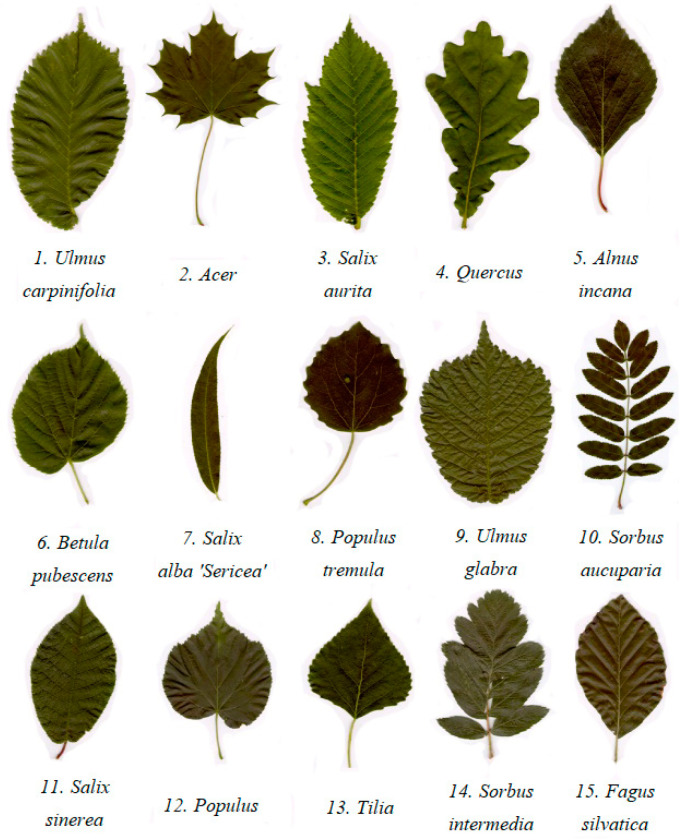
Representative leaf samples from 15 different wood species.

The Swedish Leaf Dataset [27] was adopted in our experiment, and the experimental sample consists of leaves from 15 wood species as shown in Figure 1. The 15 wood species were selected for two reasons. First, the high confidence in public available datasets is mainly due to the good scientific effectiveness of sample source and identification in public datasets, which can avoid “false identification” caused by the sameness of samples and make our experiments scientifically effective. Second, images of wood species in the same region have certain similarities in deep features, and wood species in this region are limited. For unknown wood species, there is a strong probability that they do not grow in this region, so there will be clear inconsistencies in the deep feature differences, which is more convenient for open-set recognition. There are 75 leaf images of each wood species in this dataset. All images in the original dataset were cropped and resized to 224 × 224. The configurations of the computer hardware used for conducting the experiments are presented Table 1.

### 2.2. Image Feature Extraction Based on Deep Learning Algorithm

**Figure 2:**
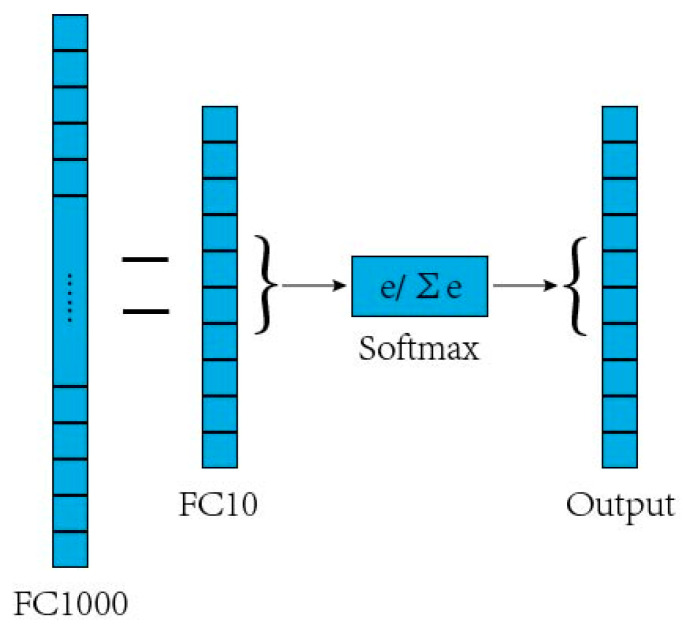
New layers appended to the deep learning model.

Due to limited images in the dataset, the CNN may suffer from overfitting by training from scratch. Therefore, it is necessary to use the transfer learning to pre-train the CNN by using part of the sample image data of the known classes (training set). The backbone model used for transfer learning is a CNN pre-trained on the ImageNet dataset. The three newly added layers (full connection layer FC10, SoftMax layer, and output layer) of the ten known tree leaf classifiers trained with ResNet50 as the trunk network are appended behind the ResNet50’s full connection layer FC1000, where the two horizontal lines represent a full connection as is shown in Figure 2. FC1000 is a feature vector with a dimension of 1 × 1000 formed by integrating features extracted from the ResNet50 network. This feature vector is the image feature obtained by the trained CNN. After the CNN is trained, partial samples of known classes and all samples of unknown classes (test set) are fed into the network, and the output features of FC1000 are used as the input for subsequent open-set recognition. In this section, CNN trained by transfer learning mechanism is used for feature extraction.

### 2.3. Weighted SVDD

The standard SVDD algorithm assumes that all samples have the same weight. But weighted SVDD assumes that the weight of “concentrated samples” is larger, while the weight of “outlier samples” is smaller. Therefore, samples close to the center of the hypersphere have larger weights, and samples far away from the center have smaller weights. In this case, the optimization problem of weight-SVDD can be expressed as Formula (1), where R is the radius of hypersphere, c is the spheroid center of hypersphere, C is the penalty coefficient, $\xi_{i}$ is the relaxation variable, respectively. $F\left(x_{i}\right)$ is the nonlinear mapping of low-dimensional samples to high-dimensional space, and the constructed Lagrange function can be expressed as Formula (2). The $\alpha_{i}\geq 0$ and $\eta_{i}\geq 0$ are Lagrangian multipliers. The partial derivatives of R, c, and $\xi_{i}$ are determined to be equal to 0 by Formula (2), and the dual form like Formula (3) can be obtained by duality principle, where $k(x_{i},x_{j})$ is the kernel function.

(1) $$\left\{\begin{array}{c} minf\left(R,c,\xi_{i}\right)=R^{2}+C\sum_{i=1}^{N}w_{i}\xi_{i}\\ s.t.\;{\;||F\left(x_{i}\right)-c||}^{2}\leq R^{2}+\xi_{i} \end{array}\right.$$

(2) $$L\left(R,c,\xi_{i}\right)=R^{2}+C\sum_{i=1}^{N}w_{i}\xi_{i}-\sum_{i=1}^{N}\alpha_{i}\left(R^{2}+\xi_{i}-{\left|\left|F\left(x_{i}\right)-c\right|\right|}^{2}\right)-\sum_{i=1}^{N}\eta_{i}\xi_{i}$$

(3) $$\left\{\begin{array}{c} \max(\sum_{i=1}^{N}\alpha_{i}k(x_{i},x_{i})-\sum_{i=1}^{N}\sum_{j=1}^{N}\alpha_{i}\alpha_{j}k(x_{i},x_{j}))\\ s.t.\;0\leq \alpha_{i}\leq Cw_{i},\;\sum_{i=1}^{N}\alpha_{i}=1 \end{array}\right.$$

Feature vectors from the FC1000 layer of the training set in Section 2.2 are used to train the weight-SVDD model, and then feature vectors outputted by the FC1000 layer of the test set samples are fed into the trained weight-SVDD model to complete the recognition of samples of known and unknown classes. In this section, a single-class classifier is used to identify images from known or unknown classes. 

### 2.4. Open-Set Recognition Framework Based on Deep Image Features

**Figure 3:**
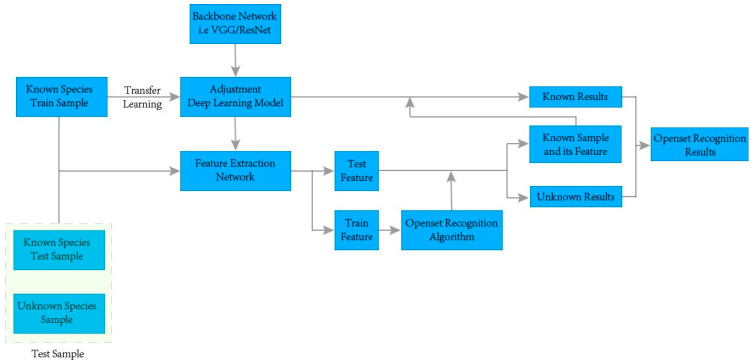
Workflow graph of our open-set recognition framework.

The recognition process of the deep image feature based on open-set recognition framework is shown in Figure 3. In the process, the following steps are required to complete the construction of the entire framework:

Step 1: Divide the dataset into known and unknown classes, and then divide the known class samples into the training set and test set. The known class training samples are used as the training set of the deep learning model and used for the open-set recognition algorithm. The total test set is composed of both the known class test samples and the unknown class test samples. The three nodes on the left side of Figure 3 are the training set of the experiment, consisting of the training samples of known classes, training samples of unknown classes, and test samples of unknown classes. 

Step 2: Rely on the transfer learning mechanism, adjust the full connection layer, SoftMax layer, and output layer, and use the known sample training set to train the CNN (such as VGG16, ResNet50, SqueezeNet, Inception V3, Densenet201, etc.). Generate feature extraction network (i.e., backbone network to FC1000 layer). In Figure 3, the backbone network nodes and the adjustment deep learning model nodes are used to complete the training of the feature extraction network.

Step 3: The training set and total test set samples are fed into a feature extraction network to generate training and test features, and the training features are used to train a traditional machine learning open-set recognition model. After the model is trained, the test features are fed into the trained model to complete the classification of known and unknown classes. In Figure 3, the train feature and test feature nodes are the advanced image features generated by the feature extraction network node on the training and test sets, and then the open-set recognition algorithm nodes are obtained by using the advanced image features from the training set.

Step 4: Re-input the generated sample features of the known classes into the deep learning model to obtain the classification results of the known classes. The final open-set recognition result can be obtained by combining the classification results of known and unknown classes. On the right-hand side of Figure 3, the samples of known classes and their features are first fed into the trained backbone network (i.e., the FC1000 features are re-input into the CNN trained by the transfer learning mechanism) to obtain the actual classes of known wood species. Then, the recognition results of known and unknown classes are combined to obtain the open-set recognition results of wood species.

### 2.5. Dataset Split and Evaluation Metric

The algorithm in this paper is based on wood leaf images, whereas current mainstream wood species recognition algorithms are mainly for color images, so correlation algorithms can be directly used to recognize this dataset. A total of 15 wood species were used in the entire experiment, each containing 75 samples. All kinds of algorithms usually adopt a 7:3/8:2 two-part partitioning method or 6:2:2/7:2:1 three-part partitioning method (for large-scale datasets, the validation set and test set are less than 20% of the total data). However, when the dataset size is small, the traditional two-part partitioning method can make a more efficient use of samples. In this paper, we use the training set to train the model, and perform the unbiased estimator directly on the test set. To test the robustness of the method, the following two datasets are split:

Case 1: Five and ten leaf wood species are selected as known classes and unknown classes, respectively. The hold-out method is used to partition the samples of known classes with a partitioning ratio of 8:2, which means that 300 training samples of known classes, 75 test samples of known classes, and 750 samples of unknown classes are all set as test samples. In total, there are 300 training samples and 825 test samples, and the ratio between known and unknown classes in the test set sample is 1:10.

Case 2: Ten and five leaf species are regarded as known classes and unknown classes, respectively. The samples of the known classes are divided by the set aside method with a partitioning ratio of 8:2, i.e., 600 training samples of the known classes, 150 test samples of the known classes, and 375 samples of the unknown classes are all set as test samples. In total, there are 600 training samples and 525 test samples, and the ratio of known class to unknown class in the test set samples is 2:5.

For quantitative evaluation, Precision and Recall are used as evaluation metrics. In addition, F1 is used to evaluate the recognition effect of known and unknown classes, and Kappa coefficient is used to evaluate the entire open-set recognition accuracy. 

Precision and Recall are defined as Equations (4) and (5). Where ${num}_{kright}$ represents the number of correctly classified samples in the known class, ${num}_{kerror}$ represents the number of incorrectly classified samples in the known class, and ${num}_{ukerror}$ represents the number of incorrectly classified samples in the unknown class. F1 can be expressed as Equation (6).

(4) $$Precision={num}_{kright}/({num}_{kright}+{num}_{ukerror})$$

(5) $$Recall={num}_{kright}/({num}_{kright}+{num}_{kerror})$$

(6) $$F1=\frac{2Precision\cdot Recall}{Precision+Recall}$$

To calculate Kappa coefficient, we first compute Pe by using Equation (7), and then compute Kappa coefficient by using Equation (8). Where classnum represents the sum of all classes of known and unknown wood species, ${num}_{i}^{t}$ represents the actual number of class i samples, ${num}_{i}^{p}$ represents the predicted number of class i samples, n represents the number of all samples, and $P_{0}$ represents the overall accuracy.

(7) $$Pe=\frac{\sum_{1}^{classnum}{num}_{i}^{t}\times {num}_{i}^{p}}{n\times n}$$

(8) $$Kappa=\frac{P_{0}-Pe}{1-Pe}$$

## 3. Results and Comparisons

### 3.1. Comparisons with Conventional Algorithms and State-of-the-Art Algorithms

**Figure 4:**
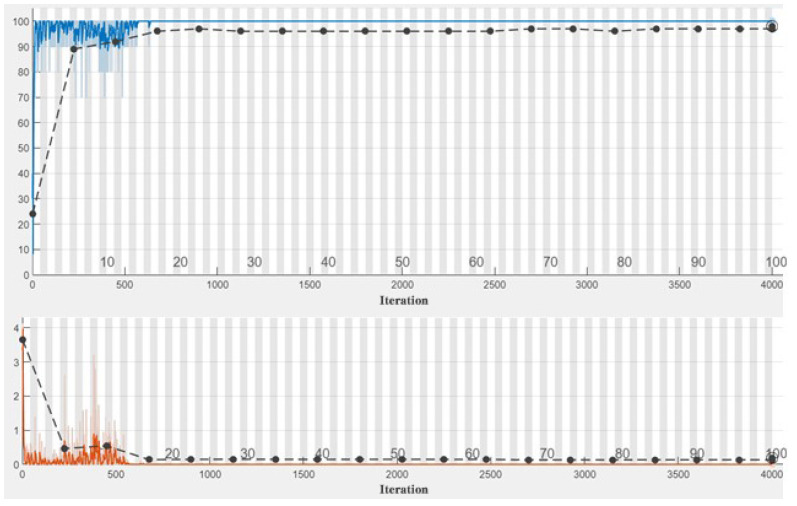
The train result of Case 2 (the blue curve is the accuracy curve, and the final validation accuracy is 98%; the orange curve is the loss curve and the final validation loss is 0.1330).

In the experiment, we used ResNet50 as the backbone network and weighted SVDD as the open-set recognition algorithm. We used the network search method [28] to obtain the optimal gamma of 0.5 and cost of 7.5. In the transfer learning process, the Resnet50 network from the Matlab Deep Learning Toolbox is used as the transfer network and the original network output layer is replaced by the new layer which is shown in Figure 2. The CNN was trained with 100 epochs. The Adam optimization algorithm [29] was used with initial learning rate 1 and the train result of Case 2 is shown in Figure 4. At present, deep learning algorithm has been used in wood species classification and has achieved good recognition effect [13,14,30]. Therefore, the experimental comparison in this paper includes both traditional machine learning classification method and advanced wood species classification algorithm. Local binary pattern (LBP) [31], histograms of oriented gradients (HOG) [32], SVM [17], ResNet50 [33], Vgg16 [34], GoogleNet [35], and SqueezeNet [36] were used for comparison.

SVM, ResNet50, Vgg16, GoogleNet, and SqueezeNet were used as classifiers. LBP and HOG were used as texture feature extraction algorithms. Similarly, wood species leaf images were fed into ResNet50, Vgg16, GoogleNet, and SqueezeNet for recognition using transfer learning. We extracted LBP and HOG features from the collected RGB images and then fed them into an SVM classifier for recognition.

The SVM algorithm uses the RBF (Radial Basis Function) kernel function, and the grid search method obtains the optimal gamma of 0.65 with a cost of 3.3. The experimental results under the setup are shown in Table 2 and Table 3. The parameters are defined in Equations (4)–(8). As can be seen from Table 2 and Table 3, neither traditional machine learning algorithms nor advanced wood species recognition algorithms can effectively perform the open-set recognition of wood species leaves, whereas our algorithm can achieve promising results.

In Table 2 and Table 3, the method columns show the method to be used. As an example, we use LBP-LibSVM, where LBP represents the front-end texture feature extraction algorithm, LibSVM represents the back-end classification algorithm, and deep learning algorithms integrate feature extraction and classification algorithms. All classes recognized by a traditional closed-set are known classes, so their output are a known sample no matter what type of sample is input. In both cases, we can see that the closed-set recognition algorithm fails to recognize known and unknown classes.

### 3.2. Compared with Open-Set Recognition Algorithms

The proposed algorithm is compared with open-set wood species recognition based on single-class classifiers, connected multi-class classifiers, OSNN [21], NCM [22], and open-max [23]. Single-class classifiers include OC-SVM, SVDD, weight-SVDD and additional algorithms, which are used to recognize species in known and unknown classes. The samples identified as known classes by the single-class classifier are then fed to the multi-class classifier for recognition, resulting in the final open-set recognition result. The second-level multi-class classifier is LibSVM. The OSNN and NCM algorithms use the output of the FC10 layer shown in Figure 2 as the feature. OSNN algorithms include two approaches: OSNN-CV (class verification) and OSNN-NNDR (nearest neighbor distance ratio). OSNN-CV judges the classification result of a test sample by determining whether the classes of the two training samples closest to the test sample agree. If so, the test sample is consistent with the class labels of the two training samples, and if not, the test sample is considered as the unknown class. OSNN-NNDR finds the train samples t and u that are closest to the test sample in both classes, computes the Euclidean distance between the test sample and the two train samples, and then computes R = dt/du. If R is less than a threshold T, the test sample is considered to be consistent with the label of the class of the nearest train sample, and if it is larger than the threshold, the test sample is considered as an unknown class. NCM computes the cluster center of the train class and then computes the Euclidean distance between the test sample and the cluster center of the train class and sets the threshold. If the distance to the nearest cluster center is less than a threshold value T, the class is identified with the nearest cluster center. If the distance is larger than a threshold value T, the test sample is considered as an unknown class. The open-max algorithm uses the output of the FC10 layer in Figure 2 as the activation vector, fits the Weibull distribution via a maximum likelihood estimation, and adjusts the output for each class using cumulative distribution function.

Feature extraction algorithms for the images used are LBP and HOG. The first-level single-class classifiers, i.e., OC-SVM, SVDD, and weight-SVDD use RBF kernel function and grid search for parameter optimization. LibSVM, a second-level multi-class classifier, uses RBF kernel function and grid search for parameter optimization as well.

Case 1: The parameters of the second-level classifier are σ=0.5 and C=4.5, and the parameters of first-level single-class classifiers are shown in Table 4. 

Case 2: The parameters of the second-level classifier are σ=0.5 and C=4.5, and the parameters of first-level single-class classifiers are shown in Table 5.

**Figure 5:**
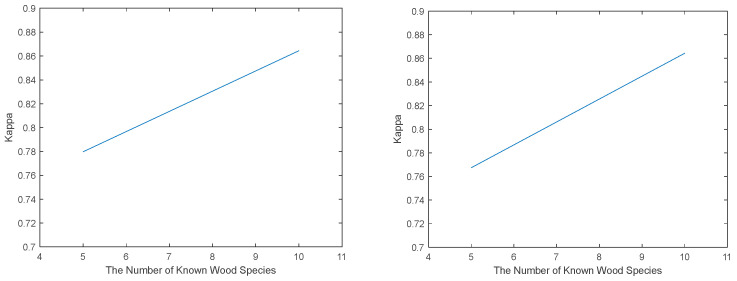
The performance improved when the known wood species increased for both Case 1 and Case 2.

The experimental results for the two cases are shown in Table 6 and Table 7. As can be seen from the tables, our algorithm achieves the best results in all six evaluation metrics in both cases. In both cases, with the number of known classes increasing, the performance of each open-set recognition algorithm used in Figure 5 improved. Case 1 is the one with the worst performance of the algorithm, mainly because the number of training samples of known classes is small (300), and the ratio between the number of test samples of known classes and that of unknown classes is too imbalanced (1:10). If a small number of unknown samples are not accurately recognized, a substantial drop in each evaluation metric is caused. Our algorithm’s performance keeps improving with the portion of test set samples increasing. Our proposed algorithm outperforms all the other open-set recognition algorithms in both cases (the proportion of known and unknown samples is 2:5).

In the left-most column of Table 4, Table 5, Table 6 and Table 7 the name of each method shows the setup of that method. For example, the LBP-weight-SVDD-LibSVM means the combination of LBP, weight-SVDD, and LibSVM. The LBP algorithm is used for feature extraction. In the two-layer recognition algorithm, the weight-SVDD is used to recognize known and unknown classes, and the LibSVM is used to recognize known wood species, respectively.

OC-SVM, SVDD, and weight-SVDD are all single-class classification algorithms with excellent performance. We found experimentally that the performance of the features extracted by the LBP algorithm combined with SVDD and other single-class classification algorithms is better than that of HOG. We conjectured that the LBP is a better fit than HOG as a feature extractor because the LBP is used as the extract feature for a low-level vision problem, while HOG is mainly used in pedestrian detection, which is a high-level vision problem. The OSNN algorithm needs to obtain the nearest training sample, and the class of the nearest training sample may be different from that of the test sample as both two OSNN algorithms show weak performance (the optimal threshold of OSNN-DDNR calculated by grid search is 0.35 and 0.37). It is difficult to obtain threshold values for NCM algorithms (the optimal threshold of NCM calculated by grid search is 0.47 and 0.53). The validity of the distance between the cluster centers of different classes and the test samples may be reduced due to the actual distribution of the test samples of different classes. The open-max algorithm uses the fully connected layers as activation vectors, uses Weibull partitioning to obtain the cumulative distribution function CDF, and corrects the classification result. However, in this paper, the implementation of the open algorithm is to perform transfer learning. After training with the Swedish Leaf Dataset, we were not able to straightforwardly verify the change of the activation vectors after tuning the network weight. But the experimental results have shown that the proposed method outperformed the traditional algorithms. The main contribution of the proposed framework is the integration of advanced image feature extraction using deep learning and the weight-SVDD algorithm. In terms of single-class recognition, the experimental results show that the advanced image features generated by CNN are better than the traditional feature extraction algorithms. The open-max algorithm may be interesting in terms of parameter tuning, resulting in poor overall recognition results. In multi-class recognition, deep learning algorithms also outperformed traditional machine learning algorithms (e.g., LibSVM). Deep learning achieves over 95% accuracy for the recognition of known classes, while LibSVM achieves an accuracy ranging from 80% to 90%.

## 4. Conclusions

In this paper, we have proposed an open-set recognition framework based on deep learning feature extraction and completed the open-set recognition of wood species. Experimental results show that the proposed method achieved promising results in recognizing wood species with an F1-Score of 0.7797 and 0.8644, mixed recognition rates of 95.15% and 93.14%, and Kappa coefficients of 0.7674 and 0.8644 in two experimental setups with different proportions of datasets. Our experimental results have shown that leaves reduce damage to the wood itself compared with other wood species recognition methods. As shown in the experiments, the recognition results were improved when the ratio between the known class samples and the unknown class samples in the test set increases from 1:10 to 2:5, suggesting that the recognition performance is better when the dataset is more balanced.

Our proposed scheme has the following advantages. First, the proposed scheme bridges the gap between open-set and wood species leaf recognition. Second, the proposed scheme uses a deep learning model as a feature extractor for advanced image feature extraction and uses the features in combination with traditional machine learning open-set recognition algorithms to perform open-set recognition with promising results. Third, both the backbone network and the open-set recognition algorithm of the proposed scheme are replaceable and can be quickly migrated to other open-set recognition applications. Moreover, the collection of leaves is particularly convenient and does not hurt the trees. The feature extraction speed and recognition speed of the deep learning network completed by training are rapid (based on the configuration of Table 1, the extraction time is within 10 ms, and the recognition speed is within 0.5 ms).

There are still issues to be addressed in this study. First, we have used a common dataset, but the small number of wood samples in this dataset, 75 per sample, makes it almost impossible to train an excellent deep feature extraction network from scratch, hence the transfer learning is used in this paper. In this case, in the absence of other publicly available datasets, the data samples we attempt to augment have to be constructed by themselves, posing a strong challenge to the scientific validity of the data. Second, in the open-set recognition approach, we use traditional machine learning methods, which are very powerful for small-scale datasets. Whether traditional machine learning methods will work or not on large-scale datasets are unknown and needs to be further evaluated, and open-set recognition algorithms based on deep learning are playing an increasingly important role. In the near future, we plan to investigate the aforementioned issues and challenges. 

## Figures and Tables

**Table 1:** Computer hardware configuration parameters.

Configuration	Model	Parameter
CPU	AMD R7-6800H	8 Core 16 thread, CPU Clock Speed 3.20 GHZ
GPU	NVIDIA GeForce RTX 3060	12 GB, GDDR6 1780 MHZ 3584 Stream Processor
RAM	Corsair Memory	16 GB, DDR5 3600 MHZ
Hard disk	Samsung	512 GB, SSD Read: 3500 MB/s, Write 3000 MB/s

**Table 2:** Open-set recognition performance comparisons with conventional algorithms and state-of-the-art algorithms in Case 1.

Method	F1-Score	$P_{0}$	Kappa
LBP-LibSVM	0.1667	8.24%	0.0810
HOG-LibSVM	0.1667	7.52%	0.0739
ResNet50	0.1667	8.85%	0.0870
Vgg16	0.1667	8.36%	0.0822
GoogleNet	0.1667	8.85%	0.0870
SqueezeNet	0.1667	9.09%	0.0894
Our Open-set Recognition Framework	0.7797	95.15%	0.7674

**Table 3:** Open-set recognition performance comparisons with conventional algorithms and state-of-the-art algorithms in Case 2.

Method	F1-Score	$P_{0}$	Kappa
LBP-LibSVM	0.4444	24.57%	0.2404
HOG-LibSVM	0.4444	23.05%	0.2254
ResNet50	0.4444	27.81%	0.2723
Vgg16	0.4444	26.29%	0.2573
GoogleNet	0.4444	27.43%	0.2686
SqueezeNet	0.4444	27.24%	0.2667
Our Open-set Recognition Framework	0.8644	93.14%	0.8644

**Table 4:** Open-set recognition classifier performance in Case 1.

Method	One-Class Classifier	
	σ	C
LBP-OC-SVDD-LibSVM	6.5	0.5
LBP-SVDD-LibSVM	0.75	0.5
LBP-Weight-SVDD-LibSVM	0.75	0.5
HOG-OC-SVDD-LibSVM	0.5	0.5
HOG-SVDD-LibSVM	0.5	0.5
HOG-Weight-SVDD-LibSVM	0.65	0.5

**Table 5:** Open-set recognition classifier performance in Case 2.

Method	One-Class Classifier	
	σ	C
LBP-OC-SVDD-LibSVM	5.5	0.5
LBP-SVDD-LibSVM	0.55	0.5
LBP-Weight-SVDD-LibSVM	0.55	0.5
HOG-OC-SVDD-LibSVM	0.5	0.5
HOG-SVDD-LibSVM	0.5	0.5
HOG-Weight-SVDD-LibSVM	0.7	0.5

**Table 6:** Open-set recognition performance comparisons with two-level open-set recognition algorithms in Case 1.

Method	F1-Score	ORA	Kappa
LBP-OC-SVDD-LibSVM	0.4923	91.39%	0.5660
LBP-SVDD-LibSVM	0.5960	91.51%	0.5899
LBP-Weight-SVDD-LibSVM	0.6538	92.61%	0.6404
HOG-OC-SVDD-LibSVM	0.1205	90.91%	0.4858
HOG-SVDD-LibSVM	0.0976	90.91%	0.4858
HOG-Weight-SVDD-LibSVM	0.1205	91.03%	0.4926
OSNN-CV	0.4055	81.82%	0.2556
OSNN-NNDR	0.4234	82.55%	0.3008
NCM	0.5333	86.30%	0.4213
Open-max	0.6509	92.12%	0.6343
Our Open-set Recognition Framework	**0.7797**	**95.15%**	**0.7674**

**Table 7:** Open-set recognition performance comparisons with two-level open-set recognition algorithms in Case 2.

Method	F1-Score	ORA	Kappa
LBP-OC-SVDD-LibSVM	0.6506	82.10%	0.6491
LBP-SVDD-LibSVM	0.7623	84.57%	0.6941
LBP-Weight-SVDD-LibSVM	0.8087	87.43%	0.7510
HOG-OC-SVDD-LibSVM	0.3333	75.43%	0.5038
HOG-SVDD-LibSVM	0.2825	75.24%	0.4960
HOG-Weight-SVDD-LibSVM	0.3957	77.33%	0.5356
OSNN-CV	0.6537	72.19%	0.4364
OSNN-NNDR	0.6538	72.37%	0.4446
NCM	0.8127	83.80%	0.6476
Open-max	0.8551	89.33%	0.7868
Our Open-set Recognition Framework	**0.8644**	**93.14%**	**0.8644**

## Data Availability

Our datasets are from the public dataset, Swedish Leaf Dataset (https://www.cvl.isy.liu.se/en/research/datasets/swedish-leaf/, accessed on 8 March 2016).
